# Artificial Intelligence in Rotator Cuff Tear Detection: A Systematic Review of MRI-Based Models

**DOI:** 10.3390/diagnostics15111315

**Published:** 2025-05-23

**Authors:** Umile Giuseppe Longo, Benedetta Bandini, Letizia Mancini, Mario Merone, Emiliano Schena, Alessandro de Sire, Pieter D’Hooghe, Leandro Pecchia, Arianna Carnevale

**Affiliations:** 1 Fondazione Policlinico Universitario Campus Bio-Medico, Via Alvaro del Portillo, 200, 00128 Rome, Italy; benedettabandini.000@gmail.com (B.B.); l.mancini@policlinicocampus.it (L.M.); m.merone@unicampus.it (M.M.); leandro.pecchia@unicampus.it (L.P.); arianna.carnevale@unicampus.it (A.C.); 2 Research Unit of Orthopaedic and Trauma Surgery, Department of Medicine and Surgery, Università Campus Bio-Medico di Roma, Via Álvaro del Portillo, 21, 00128 Rome, Italy; 3 Research Unit of Measurements and Biomedical Instrumentation, Università Campus Bio-Medico di Roma, Via Álvaro del Portillo, 21, 00128 Rome, Italy; e.schena@unicampus.it; 4 Research Unit of Intelligent Health Technologies, Department of Engineering, Università Campus Bio-Medico di Roma, Via Álvaro del Portillo, 21, 00128 Rome, Italy; 5 Department of Medical and Surgical Sciences, University of Catanzaro “Magna Graecia”, 88100 Catanzaro, Italy; alessandro.desire@unicz.it; 6 Research Center on Musculoskeletal Health, MusculoSkeletalHealth@UMG, University of Catanzaro “Magna Graecia”, 88100 Catanzaro, Italy; 7 Aspetar Orthopedic and Sports Medicine Hospital, Aspire Zone, Sportscity Street 1, Doha P.O. Box 29222, Qatar; pieter.dhooghe@aspetar.com

**Keywords:** artificial intelligence, MRI, rotator cuff, diagnosis

## Abstract

**Objective**: This descriptive systematic review aimed to assess in the available literature on the current application and overall performance of Artificial Intelligence (AI) models in the diagnosis and classification of Rotator Cuff Tears (RCTs) using MRIs. **Methods**: The systematic review was performed by two of the authors from 2020 to November 2024. Only diagnostic studies involving AI application to MRI images of the rotator cuff were considered, including supraspinatus and biceps tears. Studies evaluating AI applications to Ultrasound or X-ray, or including only healthy rotator cuffs, were not analyzed in this paper. **Results:** The coronal plane in the T2 sequence emerged as the predominant imaging protocol, with the VGG network being the most widely utilized AI model. The studies included in this research exhibited a solid performance of the AI models with accuracy, ranging from 71.0% to 100%. The statistical analysis revealed no significant differences (*p* > 0.05) in accuracy, sensitivity, specificity, or precision between AI and human experts across studies that included such comparisons. **Conclusions:** While AI can significantly improve diagnostic efficiency and workflow optimization, future studies must focus on external validation, regulatory approval, and AI-human collaboration models to ensure safe and effective integration into orthopedic imaging.

## 1. Introduction

Rotator cuff disease is the most common cause of shoulder pain, affecting 6.8% to 22.4% of patients older than 40 [1].

Calcific tendinitis, tendinosis, and tendon tears are the main causes of rotator cuff pathology [2]. Specifically, the most prevalent condition in patients over 60 is Rotator Cuff Tears (RCTs), specifically of the supraspinatus muscle, which affects 61.9% of men and 38.1% of women. [3].

The diagnosis is primarily based on both patient-reported symptoms and imaging techniques, including ultrasound, X-ray, Computed Tomography (CT), and Magnetic Resonance Imaging (MRI). The current gold standard for RCT diagnosis, prognostic feature depiction, and surgical planning is MRI [4]. Advanced fatty infiltration of the muscle and reduced acromiohumeral distance have been shown to be associated with long-standing injury to the RC and are indicative of a pre-existing tear if detected shortly after a trauma [5]. Differences in tendon kinking, muscle edema, and the degree of muscle atrophy observed on MRI have been shown to help distinguish between different types and stages of rotator cuff injuries [6].

However, the possibility of misdiagnosis can be increased by a number of circumstances. These consist of deceptive image artifacts or the existence of other diseases that could obstacle the diagnosis. As a result, studies investigating the application of computer-aided diagnostic tools to improve diagnostic accuracy and clinical decision-making have significantly increased [7].

In particular, the application of Artificial Intelligence (AI) would be particularly valuable for pathology identification, improving the diagnostic performance of medical radiologists while minimizing subjectivity and mistakes caused by inattention and fatigue [8].

Various AI applications in musculoskeletal imaging have been reported in the literature, including fracture detection, bone age estimation, osteoarthritis grading, soft tissue tumor classification, and implant analysis. These models are trained on annotated imaging datasets to identify patterns, quantify structures, or predict clinical outcomes [9].

AI can be applied through various techniques, primarily Machine Learning (ML) and Deep Learning (DL). ML involves the creation of automated computer systems that predict outcomes using mathematical algorithms. These models are developed using two types of datasets: a training set for constructing the mathematical model and a testing set for evaluating its effectiveness [10].

In contrast, DL is a more sophisticated subset of ML that enables unsupervised learning from unstructured and unlabeled data, effectively filtering out irrelevant factors during the process [11].

These processes are trained on medical databases containing big data, most of which are generated by radiomics, a quantitative method in radiology that provides clinicians with additional information using advanced mathematical analysis. By analyzing patterns, intensity, shape, and pixel relationships, radiomics quantifies textural details, offering objective, data-driven insights that complement traditional image interpretation [12].

Previous systematic reviews, such as the ones published by Zhan et al. in 2024 [7] and Rodriguez et al. in 2023 [13], have analyzed AI applications in the identification of various rotator cuff pathologies across various imaging modalities.

Similarly, Garcia et al. in 2024 [14] evaluated the performance of both DL and ML models across various imaging techniques. However, there remains a gap in the literature specifically focused on MRI-based DL diagnosis of RCTs.

This review addresses that gap by offering a comprehensive and updated evaluation of DL models applied specifically to shoulder MRI for the diagnosis and classification of RCTs. In contrast to earlier work, this review emphasizes MRI as the gold standard in imaging and explores diagnostic performance in greater detail by reporting metrics, such as accuracy, sensitivity, specificity, precision, and Dice coefficient. It also includes tear stratification data, allowing for a deeper understanding of how well models differentiate between degrees of injury severity. Additionally, the review discusses comparisons between AI and human experts, critically evaluating the imaging protocols and model architectures employed. Finally, it outlines key limitations in the current literature and offers practical recommendations for standardization and future research directions. These contributions support clinical adoption and the design of more robust, reproducible AI tools in musculoskeletal radiology.

Hence, this systematic review aims to provide an updated analysis of the diagnostic performance of MRI-based AI models for detecting and classifying rotator cuff pathologies and to assess the impact of radiomics on improving diagnostic consistency and accuracy.

## 2. Materials and Methods

### 2.1. Eligibility Criteria

The present review includes retrospective diagnostic study designs published after December 2020. Considering the authors’ proficiency in various languages, articles in English and Italian were screened. Peer-reviewed articles of each level of evidence according to the Oxford classification were included.

Only studies involving the application of AI tools to MRI images of the rotator cuff were considered, including supraspinatus and biceps tears. Studies evaluating AI applications to Ultrasound or X-ray, or including only healthy rotator cuffs, were not analyzed in this study. Systematic reviews, technical notes, letters to editors, instructional courses, or studies including pathologies different from those were excluded. Studies lacking stratified results were not considered. In vitro, animal, cadaver, and biomechanical studies were excluded.

### 2.2. Information Sources

A systematic review was performed using the Preferred Reporting Items for Systematic Reviews and Meta-analyses (PRISMA) guidelines [15]. The review protocol was not registered with PROSPERO, but it was performed in accordance with the PRISMA guidelines and registration information. Medline, EMBASE, Scopus, CINAHL, and CENTRAL bibliographic databases were searched using the following string:

((((((((((MRI) OR (magnetic AND resonance)) AND (shoulder AND joint)) AND (supraspinatus AND tendon)) OR (supraspinatus AND muscle)) AND (tear) OR (lesion)) AND (diagnostics) AND (segmentation)) AND (computer AND vision)) AND (artificial AND intelligence)) OR (deep AND learning)) AND (supraspinatus AND tear).

The search was performed by two authors from 2020 to November 2024, and articles from the inception of the database to November 2024 were searched.

Keywords were used both isolated and combined. Additional studies were searched among the reference lists of selected papers and systematic reviews.

### 2.3. Search Strategy and Data Collection Process

Data extraction was performed by two independent authors, and differences were reconciled by mutual agreement. In case of disagreement on the inclusion or exclusion of articles, a third reviewer was consulted. One author performed the review and organization of the titles in order to limit the bias.

The reviewers used the following screening approach: the title and abstract were reviewed first, then the full articles. The full text of papers not excluded was evaluated and eventually selected after a discussion between the reviewers. In case of disagreement, the third reviewer was consulted.

The initial search strategy was organized according to the PICO (Population, Intervention, Comparison, Outcome) structure. This systematic review aims to describe whether AI tools applied to shoulder MRIs of patients suspected of RCTs or other shoulder pathologies (P) performed automated diagnosis, segmentation, and classification (I) comparable to standard MRIs or radiologist diagnoses (C). The outcomes (O) assessed were: diagnostic accuracy, sensitivity, specificity, precision, and Dice coefficient.

**Figure 1:**
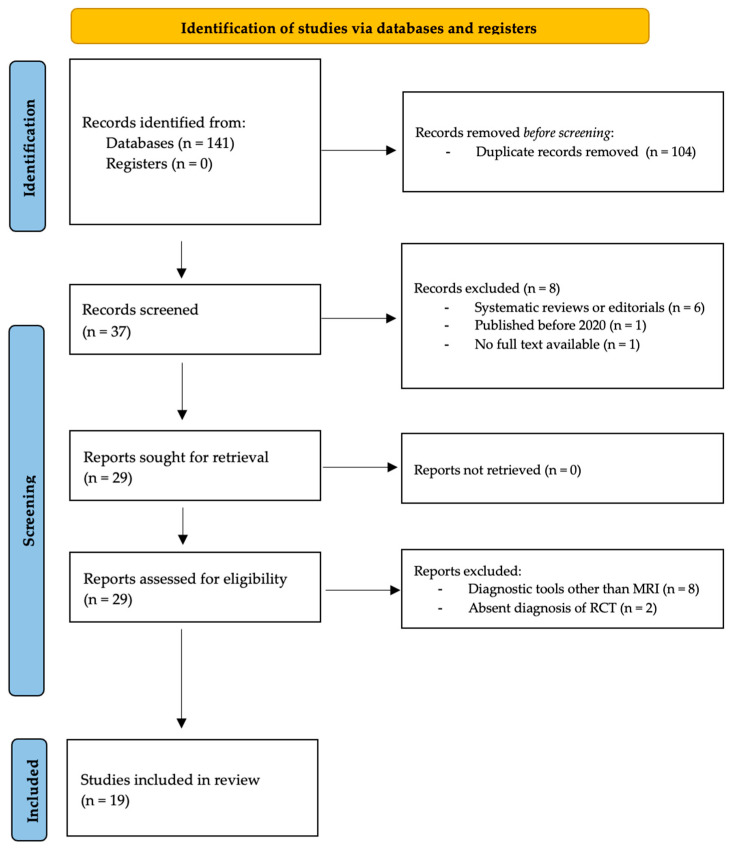
PRISMA Flowchart.

The number of articles included or excluded was registered and reported in the PRISMA flowchart. Guidelines by Moher et al. were followed to design the PRISMA chart (Figure 1) [16].

### 2.4. Data Items

The extracted data included cohort demographics, such as author, year of publication, cohort sample size, mean age, and gender distribution, as well as study specifics such as study objective and rotator cuff pathology (Table 1). Tear specifics and classification were then reported in Table 2.

General study characteristics, including MRI imaging planes, MRI sequences, and number of slices, were also recorded (Table 3).

Moreover, information regarding the AI model application was summarized in Table 4, including the AI model, the number of slices, the training sets and test sets images, and ground truth references.

Finally, diagnostic performance outcomes evaluated included percentage of accuracy, sensitivity, specificity, precision, and Dice coefficient (Table 5).

All results compatible with each outcome domain were sought, and any exclusions were based on predefined criteria related to our research questions.

### 2.5. Study Risk of Bias Assessment

Given the designs of the included studies, the quality of all included studies was assessed using the QUADAS-2 tool (https://mcguinlu.shinyapps.io/robvis/ accessed on 15 November 2024), which is designed to evaluate the accuracy of diagnostic studies [36]. Selected articles were independently evaluated by two reviewers and verified by a third in case of disagreement.

### 2.6. Synthesis Method

The synthesis of results was performed using a descriptive approach due to the high heterogeneity among the included studies, which precluded the possibility of conducting a meta-analysis. Data were extracted from each article and compiled in structured Excel spreadsheets to compare study characteristics across several domains, including cohort size, imaging protocols, AI model types, and diagnostic performance metrics (accuracy, sensitivity, specificity, precision, and Dice coefficient).

A qualitative synthesis was used to identify trends in AI model usage, commonly applied MRI sequences (e.g., T2-weighted coronal), and types of ground truth references.

When possible, a quantitative comparative analysis was also conducted for the studies that directly compared AI diagnostic performance with human experts. Paired *t*-tests were performed to compare the accuracy, sensitivity, specificity, and precision between AI and human diagnoses across those studies. A *p*-value less than 0.05 was considered statistically significant.

## 3. Results

### 3.1. Study Selection

This systematic review was conducted and reported in accordance with the Preferred Reporting Items for Systematic Reviews and Meta-Analyses (PRISMA) 2020 guidelines [15].

The literature search identified 141 articles published between 2020 and 2024. No additional studies were found in the grey literature, and no unpublished studies were retrieved. Duplicate removal resulted in the exclusion of 104 studies, leaving 37 articles for screening. Eight articles were excluded based on title and abstract (systematic reviews and editorials: *n* = 6; studies published before 2020: *n* = 1; no full text available: *n* = 1). Twenty-nine articles were screened by full text. Ten were excluded (US or X-ray-based studies: *n* = 8; absent diagnosis of RCT: *n* = 2). At the final screening, 19 articles met the selection criteria and were included in the review [17,18,19,20,21,22,23,24,25,26,27,28,29,30,31,32,33,34,35]. The PRISMA flowchart of the literature search is reported in Figure 1. Rules by Page et al. were followed in designing the PRISMA chart [15].

### 3.2. Quality of Evidence

The QUADAS-2 tool for diagnostic studies was used to assess the methodological quality of each article [36].

Out of the 19 included studies, four were identified as “low risk of bias” studies [19,24,28,30]; 13 were identified as “some concerns” studies [17,20,21,23,25,26,27,29,31,32,33,34,35], and two studies resulted in having a “high risk of bias” [18,22].

**Figure 2:**
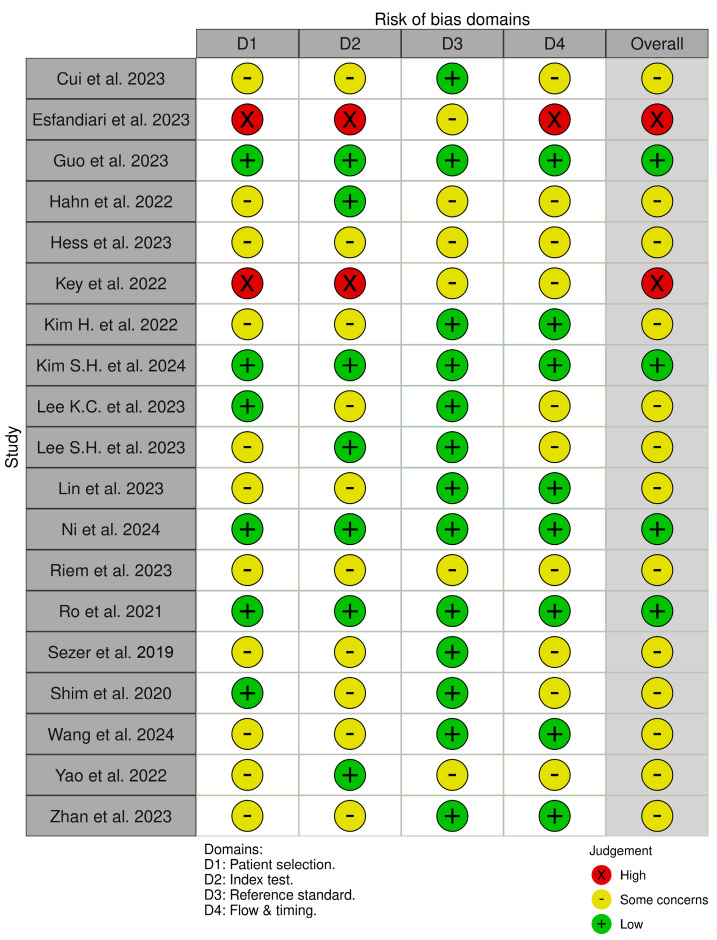
QUADAS-2 Tool Results [17,18,19,20,21,22,23,24,25,26,27,28,29,30,31,32,33,34,35].

The risk of bias assessment is reported in Figure 2. Each study is evaluated across four domains: (1) Patient Selection, (2) Index Test, (3) Reference Standard, and (4) Flow and Timing. The color coding indicates the level of bias: green for low risk, yellow for some concerns, and red for high risk. This visual summary highlights the overall methodological quality and helps assess the reliability of diagnostic accuracy results reported in the included studies.

No formal assessment of reporting bias was conducted, as the review did not include a meta-analysis, and the small number of studies limited the applicability of publication bias detection tools.

### 3.3. Cohort Characteristics

All the selected studies correctly reported the number of patients. This review included 10,277 patients. The study by Shim et al. [32] reported the highest number of patients (*n* = 2124). The study by Kim et al. [23], on the other hand, reported the lowest number of patients (*n* = 56).

The mean age and gender were not specified by all articles; however, the lowest reported age was 47.2 ± 10.0, while the highest was 64.5 ± 8.2. Additionally, most of the studies included a predominantly female cohort, with only four studies reporting a majority of male participants [21,27,29,34].

A meta-analysis was not performed at the end of the review due to the heterogeneity of the data of the selected articles. The cohort characteristics are shown in Table 1.

### 3.4. Individual Study Objectives

All studies evaluated RCTs. Specifically, nine authors studied the Supraspinatus muscle [17,19,23,27,28,30,33,34,35], while two studies focused on the Biceps Muscle [20,22]. Three studies targeted RC segmentation [21,26,33].

Finally, 16 studies focused on the diagnosis of RCT by MRI imaging [17,18,19,20,22,23,24,25,27,28,29,30,31,32,34,35]. Of these, nine studies [19,23,27,28,29,30,31,32,35] further classified the tears between partial tear, full tear, or even small, medium, large, and massive. Across all papers reporting raw counts, the dataset comprises 6721 torn tendons versus 3123 intact tendons, indicating that the AI models were generally trained and tested on tear-heavy cohorts.

The study’s objectives are summarized in Table 1, while tear classification is shown in Table 2.

### 3.5. MRI Acquisition Parameters

The most common plane of acquisition for the MRI slices was the coronal plane, employed by 17 studies [17,18,19,20,21,23,24,25,26,27,28,30,31,32,33,34,35]. Ten studies used the sagittal plane [18,20,21,24,25,26,27,28,29,32], with only one of these utilizing it exclusively [29]. The least commonly used plane was the axial plane, which was employed in only nine studies [18,20,21,22,24,25,26,30,32], with only one of these utilizing it exclusively [22].

Regarding the MRI sequences, T2 and Proton Density (PD) were the most common, employed by nine [17,20,22,23,24,25,26,27,32,34,35] and seven studies [19,24,25,27,28,31,33], with six of these utilizing it exclusively in the first [17,20,22,23,34,35] or the second [19,28,31,33]. Finally, T1 was the least frequently utilized sequence, applied only in six articles [21,24,26,29,30,32], of which three used it solely [21,29,30].

Eight articles reported the number of slices analyzed [17,19,22,24,26,27,33,34]. The study by Lin et al. [27] reported the lowest number of slices (*n* = 32). The study by Yao et al. [34], on the other hand, reported the highest number of slices (*n* = 4287).

The MRI acquisition specifics are summarized in Table 3.

### 3.6. AI Models and Learning Data

The most commonly utilized AI models were U-Net and VGG, respectively, and were applied in five [17,26,29,33,34] and four studies [22,28,30,35]. These models were implemented either independently or in combination with other models. Additionally, ResNet [17,27,34] and nnU-Net [21,24] systems were employed to analyze MRIs in three studies each, while DenseNet was employed in two studies [17,23,35]. Finally, MobileNet [18], SqueezeNet [18], Xception [19], AIR Recon [20], INCA [22], YOLO [25], RC-MTL [28], CapsNet [31], and VRN [32] were each utilized in a single article.

To ensure the accuracy of the results provided by the AI models, all studies analyzed in this research established a ground truth reference, which was verified either before or after applying the AI tool to the MRI images. A musculoskeletal radiologist was consulted by eight authors [17,21,24,25,27,33,34,35], while in six studies [18,23,26,29,30,31], the comparison was performed by an orthopaedic surgeon. Lastly, the least commonly employed ground truth reference was represented by intra-operative arthroscopic findings in five studies [19,20,22,28,32].

All information regarding the employment of the models is reported in Table 4.

### 3.7. AI Model Performance Analysis

AI model accuracy was evaluated by 13 articles [17,18,19,20,22,25,27,28,30,31,32,34,35]. The lowest accuracy was obtained by Guo et al. [19] (71.0%), while the highest value was achieved by Key et al. [22] (100%). Sensitivity was reported in 14 articles [17,18,19,20,21,22,24,25,26,28,30,32,34,35]. The lowest sensitivity was registered by Hahn et al. [20] (72.7%), whereas both Hess et al. and Key et al. [21,22] reported the highest sensitivity (100%). Specificity was analyzed by 13 articles [17,18,19,20,21,22,25,26,28,30,32,34,35], of which the highest and lowest values were obtained, respectively, by Hahn et al. and Key et al. [20,22] (100%) and by Guo et al. [19] (69.6%). Eight studies analyzed the model precision [18,19,22,24,25,26,32,33], achieving values ranging from 54.0% [19] to 100% [22].

Finally, the Dice score was evaluated by eight studies [21,23,24,26,29,30,33,34], among which Ro et al. [30] reported the highest value (0.94) while Yao et al. [34] reported the lowest value (0.81).

Four studies compared the performance of the AI model with that of an orthopedic specialist [17,19,27,32]. The *p*-values for the evaluated metrics were calculated using a paired *t*-test, comparing AI and specialist results from the same studies in terms of accuracy, sensitivity, specificity, and precision (*p* = 0.87, 0.52, 0.68, and 0.63, respectively). No statistically significant differences were observed between AI and specialists.

The AI model results are reported in Table 5.

## 4. Discussion

This descriptive systematic review aimed to assess the available literature on the current application and overall performance of AI models in the diagnosis and classification of RCTs using MRIs.

The findings reveal that AI-based models exhibit high accuracy, sensitivity, and specificity, often approaching the performance of human specialists. The studies included in this research exhibited solid performance of the DL models, with accuracy, sensitivity, specificity, precision, and Dice ranging from 71.0% to 100%, 72.7% to 100%, 69.6% to 100%, 54.0% to 100%, and 0.94 to 0.81, respectively. Several studies exceeded 90% accuracy in classification tasks. The high values for sensitivity and specificity also showed that these models successfully detect both positive and negative cases.

Notably, the studies conducted by Key et al., Ni et al., and Ro et al. obtained the highest accuracy rates (100%, 98.0%, and 99.89%, respectively) [22,28,30]. These studies also employed the same AI model, VGG. This consistency in performance across different research groups may suggest a potential superiority of the VGG model in medical image analysis. This result has also been validated by the study conducted by Saavedra et al. [37] in 2023, who trained CNN models, including VGG-19, ResNet-50, and Inception-v3, to classify supraspinatus muscle fatty infiltration using shoulder T2-weighted MRI images. The VGG-19 model demonstrated exceptional performance, achieving an accuracy of 97.3%, a sensitivity of 94.7%, and a specificity of 97.5%.

The present review also reported a direct comparison between AI models and orthopedic specialists. Statistical analysis revealed no significant differences (*p* > 0.05) in accuracy, sensitivity, specificity, or precision between AI and human experts across studies that included such comparisons. This finding supports the notion that AI can serve as a reliable decision-support tool for radiologists and orthopedic surgeons. In particular, AI’s ability to rapidly analyze MRI scans and provide quantitative assessments holds significant promise for improving diagnostic efficiency.

However, it is important to note that the overall performance of AI models is not universally consistent across studies. The observed variability in AI results underscores the significant influence of factors such as dataset quality, annotation consistency, model training approaches, and ground truth reference. For instance, only five studies used arthroscopic confirmation of RCTs as the gold standard, which is currently identified as the gold standard for RCT diagnosis [38], while others relied solely on radiologist interpretations, which can introduce subjectivity. This discrepancy in ground truth labeling may lead to inconsistent AI training and variable performance metrics. However, not all rotator cuff tears necessitate surgical intervention. Therefore, arthroscopic confirmation is not always feasible [39]

Additionally, the MRI acquisition parameters varied significantly between the included studies, affecting model reproducibility. The coronal plane in the T2 sequence emerged as the most commonly employed imaging protocol across the studies analyzed. In particular, in the present literature, the coronal plane was found to be more appropriate for detecting tendon ruptures in the shoulder when using visual descriptors such as the mean intensity value of the supraspinatus tendon [40], as this plane offers enhanced sensitivity and specificity for identifying tendon ruptures, further confirmed by other research [7,34]. This consistency suggests that the coronal T2 sequence may offer particular advantages in visualizing the structures of interest, potentially contributing to more reliable assessments. It is recommended that further studies adopt this protocol to strengthen the validity of future research and enhance the comparability of results. Standardizing the imaging approach in this manner could promote greater homogeneity within study cohorts, thereby reducing variability and ensuring more robust and generalizable findings. Thus, while AI has shown strong performance in controlled environments, these sources of variability must be addressed before AI can be seamlessly integrated into clinical practice.

This review presents points of strength. Firstly, the articles selected for analysis were published between 2020 and 2024, ensuring that the included studies reflect the most recent advancements in the field. Additionally, the studies assessed using the QUADAS-2 tool demonstrated a relatively low risk of bias, further supporting the validity of the findings.

Nonetheless, this review is subject to certain limitations.

Despite the growing interest in AI applications for rotator cuff tear diagnosis, publicly available MRI datasets specifically labeled for this purpose remain limited. Most studies included in this review relied on private, institution-specific datasets, which restricts reproducibility and external validation.

Also, the results reached from this systematic review are limited in their generalizability due to the small number of included papers, which prevented the execution of a meta-analysis and conferred this review a predominantly descriptive character. Future research should seek to increase the dataset and implement more consistent procedures to improve comparability among studies. Furthermore, the studies analyzed exhibited a high degree of heterogeneity, encompassing variations in AI algorithms, evaluation criteria, and reference standards, as well as different MRI protocols in terms of plane and sequence of acquisition, number of slices, and ground truth method. In fact, the majority of studies had the MRIs first evaluated by either experienced orthopaedic surgeons or musculoskeletal radiologists, while only a minor subgroup utilized arthroscopic findings. The latter confers more objectivity to the findings, reducing potential error in the dataset. Adopting arthroscopy or surgical findings as the reference standard would be preferred for the design of more reliable and accurate AI models. Lastly, although this systematic review adhered to the PRISMA guidelines, the relatively small number of included studies limits the generalizability of the conclusions drawn.

Future research should aim to expand the size and diversity of datasets by incorporating multi-center, multi-population imaging sources, which would improve the robustness and generalizability of AI models. In addition, adopting more standardized methodologies, such as consistent MRI acquisition protocols, uniform tear classification systems, and clearly defined ground truth references, would greatly enhance the comparability of results across studies. These efforts would not only facilitate more reliable meta-analyses in the future but also support the development of clinically deployable AI tools that can perform accurately in varied real-world settings.

## 5. Conclusions

MRI is considered the gold standard for diagnosing supraspinatus muscle tears, with the T2-weighted coronal plane emerging as the most commonly and effectively used imaging sequence for this purpose. This review found that DL models, particularly VGG-based architectures, have shown promising results in automating the detection and classification of rotator cuff conditions. The studies included in this research exhibited solid performance of the DL models, with accuracy, sensitivity, specificity, precision, and Dice ranging from 71.0% to 100%, 72.7% to 100%, 69.6% to 100%, 54.0% to 100%, and 0.94 to 0.81, respectively. Moreover, the statistical analysis revealed no significant differences (*p* > 0.05) in accuracy, sensitivity, specificity, or precision between AI and human experts across studies that included such comparisons

However, methodological limitations, dataset variability, and lack of standardization remain key barriers to clinical implementation. While AI can significantly improve diagnostic efficiency and workflow optimization, future studies must focus on external validation, regulatory approval, and AI-human collaboration models to ensure safe and effective integration into orthopedic imaging.

## Figures and Tables

**Table 1:** Study Objective and Cohort Characteristics.

Author and Year	Objective	Pathology	Cohort (*n*)	Mean Age	Gender	
					F	M
Cui et al., 2023 [17]	Diagnosis	SST	431	47.6 ± 15.1	251	180
Esfandiari et al., 2023 [18]	Diagnosis	RCT	150	NA	NA	NA
Guo et al., 2023 [19]	Classification	SST	69	NA	37	32
Hahn et al., 2022 [20]	Diagnosis	BT	110	57.6 ± 10.9	60	45
Hess et al., 2023 [21]	Segmentation	RCT	76	NA	29	47
Key et al., 2022 [22]	Diagnosis	BT	295	NA	NA	NA
Kim H. et al., 2022 [23]	Classification	SST	56	63.7 ± 9.3	32	24
Kim S.H. et al., 2024 [24]	Diagnosis	RCT	94	62.3 ± 7.5	67	27
Lee K.C. et al., 2023 [25]	Diagnosis	RCT	794	59.0 ± 11	420	374
Lee S.H. et al., 2023 [26]	Segmentation	RCT	303	64.5 ± 8.2	157	146
Lin et al., 2023 [27]	Classification	SST	518	59.4 ± 14.4	227	291
Ni et al., 2024 [28]	Classification	SST	3087	NA	1602	1485
Riem et al., 2023 [29]	Classification	RCT	232	NA	106	126
Ro et al., 2021 [30]	Classification	SST	240	NA	NA	NA
Sezer et al., 2019 [31]	Classification	RCT	1006	NA	NA	NA
Shim et al., 2020 [32]	Classification	RCT	2124	NA	NA	NA
Wang et al., 2024 [33]	Segmentation	SST	60	NA	NA	NA
Yao et al., 2022 [34]	Diagnosis	SST	200	47.8 ± 15.3	79	121
Zhan et al., 2023 [35]	Classification	SST	432	47.2 ± 10.0	251	181

SST: Supraspinatus Tear; RCT: Rotator Cuff Tear; BT: Biceps Tear; *n*: number; NA: Not Available; F: Female; M: Male.

**Table 2:** Tear Stratification.

Author and Year	No Tear	Tears						
		Tot	PT	FT	S	M	L	Ms
Cui et al., 2023 [17]	229	202	-	-	-	-	-	-
Esfandiari et al., 2023 [18]	75	75	-	-	-	-	-	-
Guo et al., 2023 [19]	26	43	3	20	8	6	6	0
Hahn et al., 2022 [20]	49	61	-	-	-	-	-	-
Hess et al., 2023 [21]	NA							
Key et al., 2022 [22]	140	155	-	-	-	-	-	-
Kim H. et al., 2022 [23]	10	46	6	0	6	14	12	8
Kim S.H. et al., 2024 [24]	94	6	-	-	-	-	-	-
Lee K.C. et al., 2023 [25]	100	694	-	-	-	-	-	-
Lee S.H. et al., 2023 [26]	NA							
Lin et al., 2023 [27]	133	385	231	154	-	-	-	-
Ni et al., 2024 [28]	456	2631	1012	1619	-	-	-	-
Riem et al., 2023 [29]	63	169	-	-	-	-	-	-
Ro et al., 2021 [30]	55	185	-	-	-	-	-	-
Sezer et al., 2019 [31]	627	379	-	-	-	-	-	-
Shim et al., 2020 [32]	764	1360	285	0	227	567	281	0
Wang et al., 2024 [33]	NA							
Yao et al., 2022 [34]	100	100	50	50	-	-	-	-
Zhan et al., 2023 [35]	202	230	100	130	-	-	-	-

Tot: Total; PT: Partial Tear; FT: Full Tear; S: Small; M: Medium; L: Large; Ms: Massive; NA: Not Available.

**Table 3:** MRI Acquisition Parameters.

Author and Year	Plane	Sequence	Slices
Cui et al., 2023 [17]	C	T2	36
Esfandiari et al., 2023 [18]	C, S, A	NA	NA
Guo et al., 2023 [19]	C	PD	64
Hahn et al., 2022 [20]	C, S, A	T2	NA
Hess et al., 2023 [21]	C, S, A	T1	NA
Key et al., 2022 [22]	A	T2	1169
Kim H. et al., 2022 [23]	C	T2	NA
Kim S.H. et al., 2024 [24]	C, S, A	PD, T1, T2	2820
Lee K.C. et al., 2023 [25]	C, S, A	PD, T2	NA
Lee S.H. et al., 2023 [26]	C, S, A	T1, T2	100
Lin et al., 2023 [27]	C, S, A	PD, T2	32
Ni et al., 2024 [28]	C, S	PD	NA
Riem et al., 2023 [29]	S	T1	NA
Ro et al., 2021 [30]	C, A	T1	NA
Sezer et al., 2019 [31]	C	PD	NA
Shim et al., 2020 [32]	C, S, A	T1, T2	NA
Wang et al., 2024 [33]	C	PD	200
Yao et al., 2022 [34]	C	T2	4287
Zhan et al., 2023 [35]	C	T2	NA

C: Coronal; S: Sagittal; A: Axial; PD: Proton Density; NA: Not Available.

**Table 4:** AI Model Specifics.

Author and Year	AI Model	Slices	Training Set (*n*)	Test Set (*n*)	Ground Truth Reference
Cui et al., 2023 [17]	U-Net ResNet DensNet	36	265	99	Musculoskeletal radiologists
Esfandiari et al., 2023 [18]	MobileNet SqueezeNet	NA	NA	NA	Orthopaedic surgeon
Guo et al., 2023 [19]	Xception	64	144	69	Arthroscopic findings
Hahn et al., 2022 [20]	AIR Recon	NA	NA	NA	Arthroscopic findings
Hess et al., 2023 [21]	nnU-Net	NA	111	60	Musculoskeletal radiologists
Key et al., 2022 [22]	VGG INCA	1169	NA	NA	Arthroscopic findings
Kim H. et al., 2022 [23]	nnU-Net	NA	34	11	Orthopaedic surgeon
Kim S.H. et al., 2024 [24]	nnU-Net	2820	84	20	Musculoskeletal radiologists
Lee K.C. et al., 2023 [25]	YOLO	NA	1511	391	Musculoskeletal radiologists
Lee S.H. et al., 2023 [26]	U-Net	100	182	61	Orthopaedic surgeon
Lin et al., 2023 [27]	ResNet	32	11,405	520	Musculoskeletal radiologists
Ni et al., 2024 [28]	VGG RC-MTL	NA	2470	309	Arthroscopic findings
Riem et al., 2023 [29]	U-Net	NA	202	30	Orthopaedic surgeon
Ro et al., 2021 [30]	VGG	NA	216	24	Orthopaedic surgeon
Sezer et al., 2019 [31]	CapsNet	NA	NA	NA	Orthopaedic surgeon
Shim et al., 2020 [32]	VRN	NA	1924	2000	Arthroscopic findings
Wang et al., 2024 [33]	U-Net	200	NA	NA	Musculoskeletal radiologists
Yao et al., 2022 [34]	ResNet U-Net	4287	160	40	Musculoskeletal radiologists
Zhan et al., 2023 [35]	DenseNet VGG	NA	332	100	Musculoskeletal radiologists

*n*: number; NA: Not Available.

**Table 5:** AI Model Performance Analysis.

Author and Year	Comparison	Accuracy (%)	Sensitivity (%)	Specificity (%)	Precision (%)	Dice
Cui et al., 2023 [17]	AI	92.9	91.8	94.0	NA	NA
	H	90.9	91.8	90.0	NA	NA
Esfandiari et al., 2023 [18]	-	92.6	91.7	92.2	91.1	NA
Guo et al., 2023 [19]	AI	71.0	73.9	69.6	54.0	NA
	H	86.2	93.5	82.6	72.9	NA
Hahn et al., 2022 [20]	-	88.9	72.7	100	NA	NA
Hess et al., 2023 [21]	-	NA	100	94.0	NA	0.91
Key et al., 2022 [22]	-	100	100	100	100	NA
Kim H. et al., 2022 [23]	-	NA	NA	NA	NA	0.83
Kim S.H. et al., 2024 [24]	-	NA	93.3	NA	91.2	0.92
Lee K.C. et al., 2023 [25]	-	96.0	98.0	91.0	98.0	NA
Lee S.H. et al., 2023 [26]	-	NA	97.1	95.0	84.9	0.94
Lin et al., 2023 [27]	AI	81.0	NA	NA	NA	NA
	H	79.0	NA	NA	NA	NA
Ni et al., 2024 [28]	-	98.0	96.0	93.0	NA	NA
Riem et al., 2023 [29]	-	NA	NA	NA	NA	0.92
Ro et al., 2021 [30]	-	99.8	93.3	99.9	NA	0.94
Sezer et al., 2019 [31]	-	94.7	NA	NA	NA	NA
Shim et al., 2020 [32]	AI	87.5	92.0	86.0	94.0	NA
	H	79.8	89.0	61.0	79.0	NA
Wang et al., 2024 [33]	-	NA	NA	NA	99.2	0.90
Yao et al., 2022 [34]	-	81.4	85.0	85.0	NA	0.81
Zhan et al., 2023 [35]	-	76.4	79.2	74.3	NA	NA

AI: Artificial Intelligence; H: Human; NA: Not Available.

## Data Availability

The data presented in this study are available upon request from the corresponding author.
